# Foot orthoses for flexible flatfeet in children and adults: a systematic review and meta-analysis of patient-reported outcomes

**DOI:** 10.1186/s12891-022-06044-8

**Published:** 2023-01-07

**Authors:** Leonoor N. T. Oerlemans, Charles M. M. Peeters, Roelina Munnik-Hagewoud, Ingrid M. Nijholt, Adhiambo Witlox, Cees C. P. M. Verheyen

**Affiliations:** 1 Department of Orthopaedics, Isala Hospital, Dokter van Heesweg 2, 8025 AB Zwolle, The Netherlands; 2 Department of Orthopaedics, University Medical Center of Groningen, Groningen, The Netherlands; 3 Department of Innovation and Science, Isala Hospital, Zwolle, The Netherlands; 4 Department of Radiology and Nuclear Medicine, Isala Hospital, Zwolle, The Netherlands; 5 Department of Orthopaedics, Maastricht University Medical Centre, Maastricht, The Netherlands

**Keywords:** Foot orthoses, Symptomatic flexible flatfeet, Children, Adults, Meta-analysis, Systematic review

## Abstract

**Background:**

This systematic review and meta-analysis examined the effectiveness of orthoses for flexible flatfeet in terms of patient-reported outcomes in children and adults.

**Methods:**

EMBASE, Medline (OvidSP), Web-of-Science, Scopus, CINAHL, Cochrane Central Register of Controlled Clinical Trials, i.e., Cochrane Central and Pubmed were searched to identify relevant studies since their inception up to February 2021. We included randomized controlled trials (RCT) and prospective studies in which patient reported outcomes at baseline and follow-up in an orthoses group were compared with a no orthoses or sham sole group. Methodological quality of the studies was assessed using the Revised Cochrane risk-of-bias tool for randomized trials (RoB 2) and the Risk Of Bias In Non-Randomized Studies of Interventions (ROBINS-I). A meta-analysis was performed where there were multiple studies with the same outcome measures, which was the case for the Visual Analogue Scale (VAS) for pain in adults.

**Results:**

In total nine studies were included: four RCT in children (*N* = 353) and four RCT and one prospective study in adults (*N* = 268) were included. There was considerable heterogeneity between studies. A meta-analysis demonstrated that pain reduction between baseline and follow-up was significantly larger in the orthoses (*N* = 167) than in the control groups in adults (*N* = 157; − 4.76, 95% CI [− 9.46, − 0.06], p0.05).

**Conclusion:**

Due to heterogeneity in study designs, we cannot conclude that foot orthoses are useful for flexible flatfoot in children and adults. However, based on the meta-analysis orthoses might be useful in decreasing pain in adults.

The authors did not receive support from any organization for the submitted work.

**Supplementary Information:**

The online version contains supplementary material available at 10.1186/s12891-022-06044-8.

## Introduction

Flatfoot is a usually asymptomatic condition of a lower medial arch of the foot. In literature, different definitions are used, and yet, there is no universal accepted definition. There are two types of flatfeet: flexible and rigid. In contrast with flexible flatfeet, the rigid type is less common. Rigid flatfeet account for less than 1% of the population [1], are characterized by a lowered arch both weightbearing and non-weightbearing [2], and foot orthoses probably have little effect because of the limited range of motion. The rigid deformity is outside of scope in this study.

Flatfoot can be influenced by multiple congenital and acquired factors [3]. The etiology differs between children and adults. Normal development of the foot in children is associated with physiological flatfoot [4]. Most babies are flatfooted and the arch elevates spontaneously in the first decade [5]. Flatfeet are identified as present in 54% of 3-year-olds and 26% of 6-year-olds [1]. `Children have a flatter foot structure due to immaturity of complex bone, soft tissue and neurological function which reduces over the first decade of life [6]. Prevalence in older adults has been reported to reach 19% [7].

Foot orthoses are frequently prescribed in daily practice for symptomatic flexible flatfeet. However, some studies suggest that using orthoses for asymptomatic flexible flatfeet is unnecessary [1], reporting that whilst 10% of American children with flatfeet are treated with orthotics, only 1–2% were shown to be symptomatic [1]. Adults with symptomatic flexible flatfeet are frequently treated with foot orthoses [8–11]. However, in multiple systematic reviews the literature on the effect of foot orthoses for symptomatic flatfeet is controversial, likely due to the high heterogeneity between studies [8, 12, 13]. Despite the concerns regarding the efficacy of foot orthoses for flexible flat feet, it is understood that people with flat feet can have a functional deficit. Compared to normal feet, children with flexible flatfeet score lower quality of life. This is seen in an prospective, observational study [14]. It is seen that even though parents may overestimate the severity of their child’s impairment, children with flexible flatfeet do have significantly impaired quality of life-score when compared to children with normal feet [15]. Thereby, results of another study showed significant differences in improvement between asymptomatic versus symptomatic flatfeet in children in terms of kinematics [16].

It is important to establish the effectiveness of orthoses because the large number of orthosis prescriptions worldwide has a major impact on healthcare costs. In Germany e.g., about five million people, which is about 8% of the population, were prescribed foot orthoses on indication. Consequently, in the year 2019, the costs for the statutory health insurance increased by 466.6 million euros, just because of these prescriptions [17, 18]. Moreover, evidence of a potential positive or negative effect of orthoses on flatfeet can be used in the orthopedic clinic as an argument to improve treatment of patients with flatfeet.

In previous systematic reviews, kinematic and radiological measurements were mainly used as outcome measures [13, 19, 20]. Some systematic reviews included studies without a control group or patients without symptoms or without follow-up [17, 21, 22]. One systematic review included stage 1 adult-acquired flatfoot, which is a precursor to visible changes in foot alignment [23]. In order to obtain valid evidence on the clinical effectiveness of orthoses from a patient perspective and subsequently improve evidence-based medicine in routine orthopedic practice for both children and adults with symptomatic flatfeet, a systematic examination of studies reporting patient-reported outcome measures (PROMs) that includes an appropriate control group is warranted. Our review is not the first systematic review relating to flatfoot to focus on PROMs rather than objective measures. This is, however, the first systematic review including only studies about patients with flexible flatfeet where patient-reported outcomes were measured at both baseline and follow up moments, and a control group without orthoses or sham soles was included.

This systematic review and meta-analysis examines the effect of orthoses for symptomatic flexible flatfeet in terms of PROMs in children and adults. By focusing on PROMS, this systematic review includes broader aspects of health, as described by the ICF framework, than the previous studies which focused on body structures and functions (i.e. radiological and kinematic outcome [13, 20, 24]). Moreover, many clinicians prescribing foot orthoses are more likely to use PROMs and pain scales, than radiological and kinematic outcomes [25, 26] Thus, the results of this review will be more useful for these clinicians.

## Methods

We adhered to the standard guidelines of Preferred Reporting Items for Systematic Reviews and Meta-Analyses (PRISMA) [27]. The protocol for this systematic review with meta-analysis was registered in Research Square, but not published in a peer-reviewed journal. There were no deviations from an a prior protocol throughout the methods.

### Inclusion and exclusion criteria

Randomized controlled trials and prospective studies were included if the study (1) compared orthoses in adults or children with sham soles or no orthoses in both male and female adults or children with flexible flatfeet. For children, all ages (0–18 years) were included; (2) evaluated any patient-reported outcome at baseline and after treatment with a follow-up of at least 2 weeks as the minimum follow-up deemed reasonable to determine any effect of wearing orthoses. All patient-reported outcomes that addressed the effectiveness of orthoses for flexible flatfeet and were measured at two different time points before and after intervention were considered eligible; (3) had full text available; (4) was published in English, Dutch or German. Studies concerning patients with neuromuscular or systemic diseases were excluded. Studies comparing with flatfeet with tape were excluded. Dissertations, master theses, abstracts from conference proceedings, commentaries, comments, editorials, case reports, reviews, letters, guidelines and protocols were also excluded.

### Information sources

Electronic databases (EMBASE, Medline (OvidSP), Web-of-Science, Scopus, CINAHL, Cochrane Central Register of Controlled Clinical Trials, i.e., Cochrane Central. and Pubmed) were systematically searched for relevant studies since their inception up to February 2021. In addition, reference lists of included articles were screened for eligible studies that were not found in the electronic databases. We used the mean differences in VAS-score, the information was taken per article to calculate the mean differences. In some studies the mean differences in VAS was presented. Some studies (see table 1 column ‘outcome measurement’ used other outcomes for pain, for example, yes/no in Whitford 2007, we could not use these outcomes for our calculation). Article titles, keywords, and abstracts were searched for the following keywords and their synonyms: flatfoot AND orthoses. All search strategies in the databases are specified in Supplementary data 1.

**Table 1:** Overview of study characteristics

	First author, year published	Country of conduction	Design, type of analysis	Overall risk of bias ^c^	N (% male)	Mean age in years ± SD (inclusion age range per study)	Included patients (% flexible)	Measurement method for including pes planus	Brand or type of orthoses versus control group (N)	Outcome measurement ^a^	FU	Conclusion of authors ^a^
Children	Asgaonkar 2012 [28]	India	RCT, PP	High	60 (50%)	I: 9.4 ± 2.7 (NA) C: 9.3 ± 2.4	Flatfoot (100%)	Staheli arch index Instep footprint	I: Valgus pad, rubber (30) C: No orthoses (30)	Pain (VAS)	1Y	Significantly less pain established in orthoses group compared to control group (*p* < .05)
	Hsieh 2018 [29]	Taiwan	RCT, ITT	Low	52 (54%)	I: 6.9 ± 0.6 (3–10) C: 6.2 ± 0.4	Symptomatic flatfoot (100%)	Beighton > 4 NDT ≥6 mm FPI-6 > 6 X-ray angles	I: Thermoplastic insoles with medial arch support High-density EVA (26) C: No orthoses (26)	Physical function Questionnaires: PODCI and HRQoL	3 M	Significant improvement in pain/comfort and physical health (*p* < .05) in intervention group compared to control group
	Sinha 2013 [30]	India	RCT, PP	High	81 (59%)	I: 8.3 ± 4.3 (3-17^b^) C: 8.3 ± 4.4 (3-16^b^)	Symptomatic flatfoot (100%)	Pain Fatigue Gait disturbances	I: Medial arch orthoses (55) C: No orthoses (26)	AOFAS score	>2Y	Significant improvement in AOFAS scores between intervention and control group, benefitting the intervention group (*p* < .05)
	Whitford 2007 [31]	Australia	RCT, PP	Low	160 (53%)	I: 9.5 ± 1.5 (7–11) I: 9.8 ± 1.3 C: 9.5 ± 1.4	Excess pronation (100%)	RCSP > 4 NDT	I: Custom orthoses (52) I: Ready-made orthoses (54) C: No orthoses (54)	Pain yes/no Self Perception Profile for Children	1Y	No significant differences between groups in self-perception and pain
Adults	Andreasen 2013 [32]	Denmark	RCT, PP	High	70 (19%)	43.0 ± 3.0 (18–70)	Flatfoot and chronic pain conditions in the foot (100%)	Calcaneal valgus > 6° Overload-related pain under knee	I: Medial arch and calcaneus support, EVA (20) I: Exercise program (19) I: Orthoses + exercise program (17) C: Folder with exercises (14)	Pain during walking, running, resting (VAS)	1Y	No significant differences in pain between orthoses groups compared to non-orthoses groups
	Esterman 2005 [33]	Australia	RCT, ITT, PP	High	47 (94%)	21.6 ± 3.9 (NA)	Air Force recruits with flatfeet (100%)	Arch index = footprint B/(A + B + C)	I: Flexible/semi-rigid orthoses, AOL, with rearfoot varus wedge (25) C: No orthoses (22)	Pain yes/no General foot health, injury Quality of life Physical health	8 W	No significant differences between study groups for pain, injury, foot health, and quality of life
	Shih 2011 [34]	Taiwan	RCT, PP	High	24 (75%)	I: 31.3 ± 8.3 (NA) C: 34.4 ± 9.8	Flatfoot in runners with pain over anterior knee or foot region during running (100%)	Navicular Drop Test Non-weightbearing rearfoot varus > 5° or weightbearing calcaneal valgus > 5°	I: Wedged foot orthoses, EVA (12) C: Sham orthoses (12)	Pain (VAS) after 60-minute treadmill test	2 W	Significantly less pain in the immediate and short-term effects on incidence of pain between groups (*p* < .05)
	Taspinar 2017 [35]	Turkey	PS, PP	Some concerns	60 (25%)	50.5 ± 9.0 (15–65)	Bilateral flatfoot, identical type (97%)	Navicular Drop Test X-ray: CP < 20	I: Medial arch support (20) I: Thomas heel, external support (20) C: Exercise program (20)	Foot pain index Foot function index SF36-PCS	3 M	All groups improved on pain, foot function index, and quality of life (*p* < .05); no significant difference between groups
	Yurt 2019 [36]	Turkey	RCT, ITT	Low	67 (42%)	I: 21.7 ± 2.9 (18–45) I: 23.1 ± 5.5 C: 21.1 ± 2.0	Foot pain for at least 1 month due to flatfoot (100%)	MFPI ≥6 Tibiocalcaneal angle > 5°	I: CAD-CAM orthoses and exercise (22) I: Conventional orthoses and exercise (22) C: Sham orthoses and exercise (23)	Pain (VAS) Foot function index SF-36 Satisfaction	8 W	Pain intensity and physical health significantly improved in CAD-CAM and conventional orthoses compared to sham group (*p* < .05)

^a^Only PROMs are described with corresponding conclusions in this table

^b^Only Sinha et al., presented the exact age range

^c^ Overall risk of bias assessment (high, low, some concerns) according to the RoB 2 and ROBINS I quality assessment tools presented in Figure

Abbreviations: *AAFD* Adult-acquired flatfoot deformity, *AFO* Molded angle-foot orthosis, *AOFAS* American Orthopaedic Foot and Ankle Society, *AOL* Australian orthotics laboratory, *C* Control group, *CAD-CAM* Computer-aided design/computer-aided manufacturing, *CSE* Central stabilizer element (customized orthoses), *EVA* Ethylene-vinyl acetate, *FHSQ* Foot health status questionnaire, *FU* Follow-up, *HRQoL* Health-related quality of life, *I* Intervention group, *ITT* Intention to treat, *(M) FPI* (modified) foot posture index, *M* Months, *N* Number of patients, *NA* Not available, *NDT* Navicular drop test, *PCI* Physiological cost index, *PODCI* Pediatric outcomes data collection instrument for pain/comfort and happiness, *PP* Per protocol, *PS* Prospective study, *PTTI* Posterior tibial tendon insufficiency, *QA* Quality assessment, *RCSP* Resting calcaneal stance position, *RCT* Randomized controlled trial, *SD* Standard deviation, *SF-36* Short-Form Health Survey, *UCBL* Orthosis type of University from California Biomechanics Laboratory (aims to limit motion of subtalar joint), *VAS* Visual Analogue Scale, *W* Week, *Y* Year

### Study selection

**Fig. 1:**
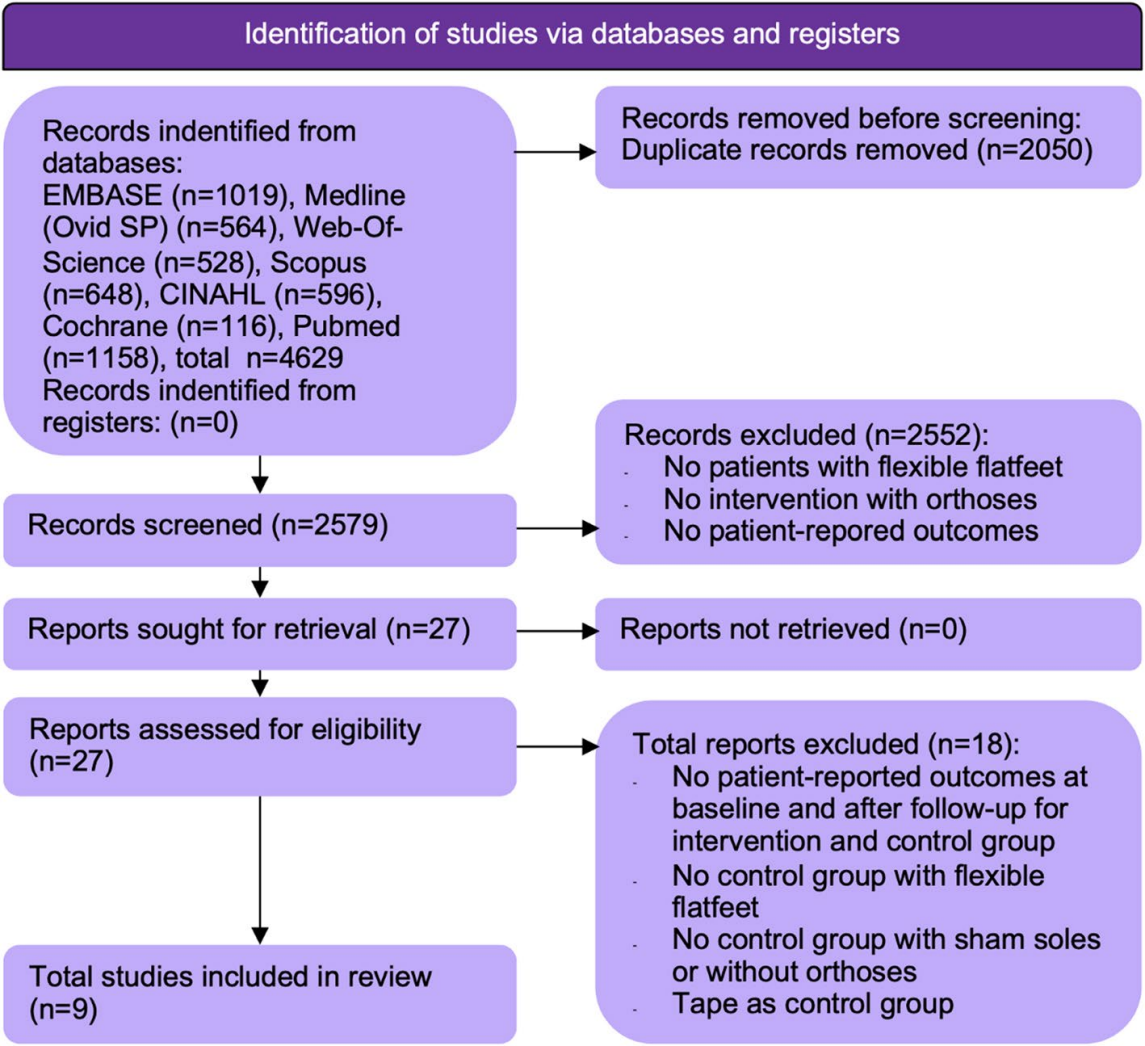
Study selection. Nine studies were included in the systematic review, four of them on children and five on adults

The information specialist of our hospital supervised performing the search. One reviewer (NO) conducted the searches (using Refworks Proquest)**,** examined article titles and abstracts for eligibility and screened full texts of potential studies to determine final eligibility for inclusion in this review and meta-analysis (Fig. 1). Uncertainty concerning inclusion of studies was solved in a single consensus meeting. Secondly, all included studies were reviewed for agreement by a second reviewer (CP).

### Risk of Bias assessment

**Fig. 2:**
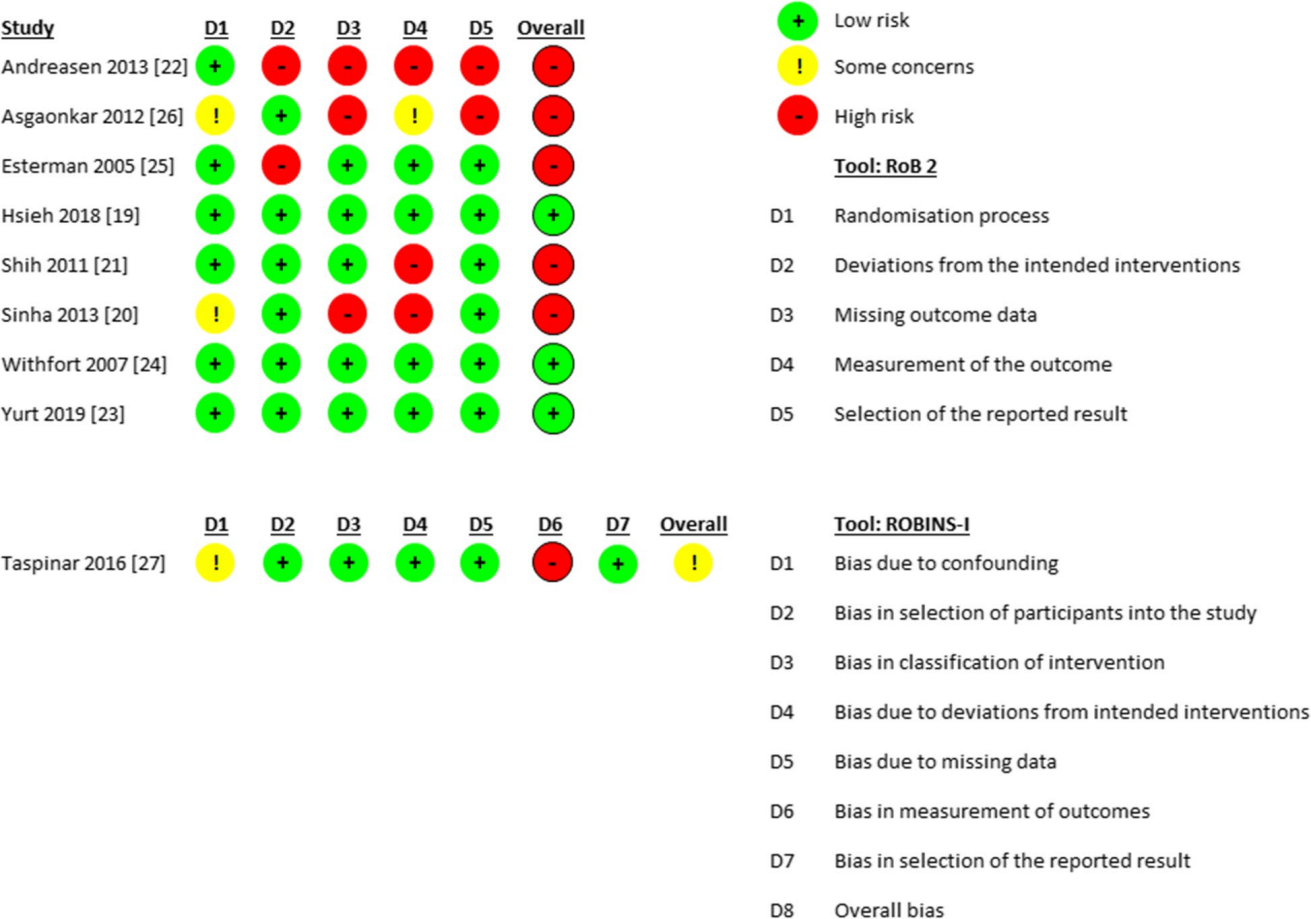
Quality assessment domains. RCTs are evaluated with the Cochrane RoB tool and the prospective study is evaluated with the Cochrane ROBINS-I tool

Two authors (NO and CP) independently assessed the methodological quality of each included study using the Revised Cochrane risk-of-bias tool (RoB 2) for randomized trials and the Risk Of Bias In Non-Randomized Studies - of Interventions (ROBINS-I) for prospective cohort studies [37, 38]. All quality criteria domains were rated as “low risk”, “some concerns”, or “high risk” by answering the corresponding questions and following the algorithm for judgment (Fig. 2). Disagreements were solved by consensus. In case of persistent disagreement, a third reviewer was consulted (IN).

### Data extraction

One author (NO) extracted the data of the included studies in Refworks Proquest. Information was collected on study design, study population, measuring method for pes planus, types of orthoses, PROMs, follow-up period, and results.

### Presentation

Meta-analyses using a random-effects model was performed if there were multiple articles – or subgroups within articles – in which the same PROM was used [39]. Outcome data of children and adult articles were not combined in the meta-analysis because of the difference in etiology of flexible symptomatic flatfeet.

When standard deviations were not provided in the article, the range split by four was used as standard deviation [40]. The I^2^ statistic was calculated to determine the percentage of variation across studies that is due to heterogeneity rather than chance. Data analysis was performed with Review Manager (Revman) version 5.4.1 [39]. *P* ≤ 0.05 was considered significant.

## Results

### Study inclusion

The initial search identified 4629 potentially relevant studies. After removing duplicates, titles and abstracts of 2579 articles were screened (Fig. 1). A total of nine studies met all eligibility criteria: four RCTs in children and four RCTs and one prospective study in adults (Table 1) [28–36]. In supplementary data 2, a reference list of all studies that underwent full-text review but were excluded, including the reasons for exclusion after each references, is presented.

### Risk of bias in studies

Three RCTs were classified as low risk of bias, five RCTs as high risk of bias. The only prospective study was qualified as “some concerns” (Fig. 2). The methodological shortcomings of the RCTs mainly concerned domain 4 (measurement of the outcome). Domain 1 (randomization process) was scored best with six studies as “low risk” and two studies as “some concerns”, followed by domain 2 (deviations from the intended interventions) and domain 5 (selection of the reported results), where six studies scored “low risk” and two studies were judged as “some concerns”.

### Study characteristics

The sample size of the included studies on the effectiveness of orthoses for flatfeet in children ranged from 52 to 160. Studies on the effectiveness in adults had sample sizes of 24 to 70 participants. Mean age of children and adults per study ranged from 6.2 to 9.5 years (weighted mean 8.8) and 21.1 to 50.5 years (weighted mean 34.8), respectively. The percentage of male in the studies about adults ranged from 19 to 94%, whereas male percentage in the studies about children ranged from 50 to 59%. Patient recruitment varied widely between the child studies (Table 1). One study recruited children in primary and secondary school [28], one in a rehabilitation outpatient clinic [29], another in an orthopedic outpatient clinic [30], and the last child study recruited among the general population via media and pamphlets [31]. Only one of these studies assessed patient compliance. Compliance with the treatment protocol was reported to be nearly 100% and was similar between the intervention and the control group [31]. Four adult studies recruited patients in outpatient rehabilitation clinics [33–36] and one study recruited patients in an outpatient orthopedic clinic [32]. The study populations included in the adult studies varied: three studies included the general patient population with symptomatic flexible flatfeet [32, 35, 36], the other two included only Air Force recruits [33] or runners [34]. Most studies specifically included patients with symptomatic flatfeet (Table 1). Only one of the adult studies measured patient compliance and reported that only half of the subjects in the orthoses group wore their orthosis most of the time or always [33]. Methods of measuring flatfeet varied between studies; most researchers used arch indexes, the Navicular Drop Test or the (Modified) Foot Posture Index as diagnostic method. The follow-up in the studies with children ranged from 3 months [29] to at least 2 years [30], and in the studies with adults from 3 weeks [34] to 1 year [32].

None of the child studies used sham soles as control group, whereas two out of five adult studies did [34, 36]. No other studies had insoles as control group. The nine studies had in total eleven intervention groups with orthoses, since there were two adult studies with both prefabricated orthoses and customized orthoses groups [31, 36]. These were all medial wedged internal orthoses. One child study and one adult study had an intervention group that used prefabricated orthoses [31, 36]. Three child studies and two adult studies had an intervention group that used custom-made orthoses [29–32, 36], of which one adult study used Computer Aided Design-Computer Aided Manufacturing (CAD-CAM) to design the orthoses [36]. One child study and three adult studies did not specify whether the orthosis were prefabricated or customized [28, 33–35]. Materials for orthoses were thermoplastic [30, 31, 33, 35], ethylene-vinyl acetates (EVA) [29, 32, 34, 36], and rubber [28].

Only one study controlled the analysis of orthoses’ effectiveness for physiotherapy as a potential confounder by splitting both the orthoses group and the no-orthoses group into a physiotherapy and no-physiotherapy group [32].

### Patient-reported outcome measures in children

Effectiveness of orthoses was mainly measured in terms of pain reduction. In one RCT it was shown that the orthoses group experienced significantly less pain, expressed as VAS score reduction over a follow-up period of 1 year, compared to no orthoses (*p* < 0.05) [28]. Step length, physical cost index (PCI), stride length, cadence, and velocity were also measured in addition to pain. An improvement in walking efficiency was seen based on these parameters.

In another RCT, that used the American Orthopaedic Foot and Ankle Score (AOFAS) for patients’ forefoot, midfoot, and hindfoot as outcome measure, the orthoses group also had significantly less pain during 2 years follow-up in all foot areas (all *p* < 0.01), whereas the no-orthoses control group only showed significantly reduced pain in the forefoot [30]. A significant difference between pain reduction in the orthoses and control groups was reported for the midfoot and forefoot (*p* < 0.05). Multiple angles were measured on X-ray. A correlation between the calcaneal pitch angle and the lateral talocalcaneal angle with the AOFAS hindfoot score was found.

When motor proficiency, presence of pain (yes/no), exercise efficiency (measured as maximal oxygen uptake by VO_2_ max), and self-perception were used as primary outcome measures in a study with customized, prefabricated orthoses and no-orthoses control groups, no difference between these groups was observed after 3 months and after 1 year follow-up [31]. In this RCT, the authors used the Self Perception Profile for Children because of the suggestion that foot orthoses might be embarrassing for children.

In the fourth RCT, physical activity (10-m normal and fast walking, stair ascent, stair descent, and chair rising), physical function, and psychometric properties (Pediatric Outcome Data Collection Instrument for evaluating pain/comfort and happiness and Pediatric Quality of Life Inventory) were evaluated at baseline and at 3 months follow-up. The intervention group showed significant improvement in all outcomes compared to the no-orthoses group (*p* < 0.05) [29].

### Patient-reported outcome measures in adults

Four RCTs and one prospective study in adults were included in this systematic review. Three studies used visual analogue scale (VAS) scores as outcome measure. One RCT included patients with excessive pronation and chronic foot pain (mean duration of pain = 7.2 years) [32]. Participants were randomized into an orthoses group, an exercise program group, an orthoses with exercise program group, and a control group, which received a folder with exercises. Pain intensity was assessed during resting, walking, and running. There was significant pain reduction during walking within all four groups between baseline and at 4 and 12 months follow-up. No significant differences could be found between the groups [32].

Another RCT examined the immediate and short-term effects of foot orthoses during a 60-minute running test in pronated-foot runners with overuse knee or foot pain during running [34]. VAS score decreased significantly in the orthoses group after a 2-week treatment (*p* < 0.01) but did not decrease in the sham sole group.

An 8-week follow-up study with three groups – CAD-CAM, conventional, and sham soles – also reported significantly less pain using VAS in both orthoses groups after treatment compared to the sham soles group (*p* < 0.05) [36]. All groups scored significantly higher on physical health (SF-36). The mental health domain of the SF-36 did not show a significant difference between the groups. The Foot Function Index (FFI) showed significantly better outcomes for the conventional orthoses group (*p* < 0.001) compared to the sham sole group.

The study population of the fourth RCT consisted of Air Force recruits. In this study population there was no significant difference in “lower limb pain in the previous 24 hours”, nor in the questionnaires General Foot Health (GFHQ), Quality of life (WHOQOL), or Physical health (WHOQOL) between the orthoses and the no-orthoses groups [33].

The only prospective study included found a significant improvement in the orthoses group, external shoe modification group, and pes planus exercise group after treatment of 3 months in terms of foot pain, FFI, and quality of life (*p* < 0.05). There were no differences between the groups [35].

### Meta-Analysis: Visual Analogue Scale

**Fig. 3:**
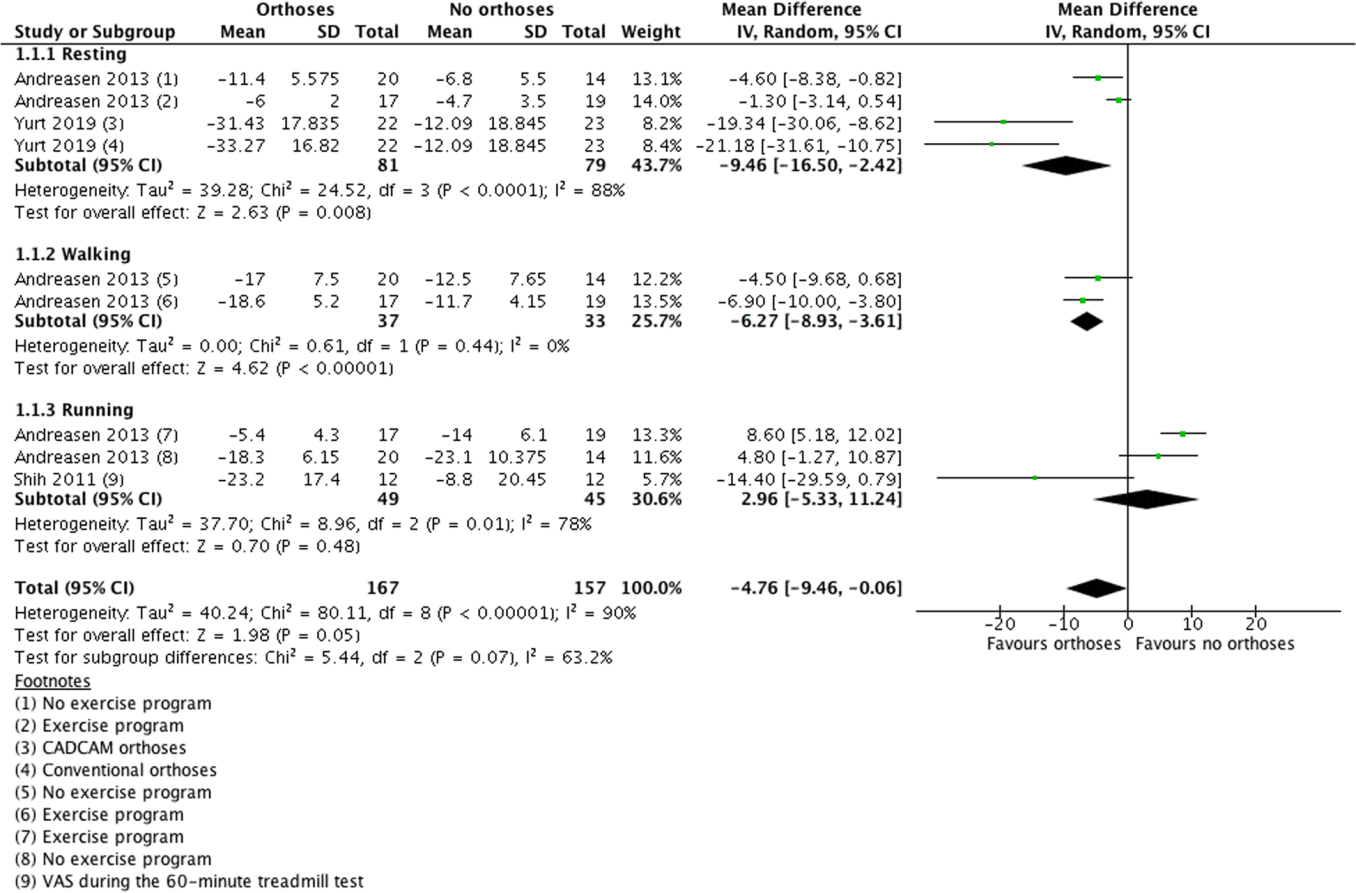
Pain outcomes (VAS) reported when comparing foot orthoses vs no orthoses/sham insoles group in adults and children with flexible flat feet. Pain reduction between baseline and follow-up in the orthoses groups was significantly larger than in the control groups during resting and walking as well as between all groups. No significant difference was seen in the running subgroup

Because of the heterogeneity in outcome measures used in the included studies, we could only perform a meta-analysis of the three adult RCTs that used VAS scores as outcome measure (Fig. 3). Analyses were stratified for the conditions in which VAS scores were measured: resting, walking, and running. Pain reduction between baseline and follow-up in the orthoses groups was significantly larger than in the control groups when resting (− 9.46, 95% CI [− 16.50, − 2.42], *p* < 0.001) and when walking (− 6.26, 95% CI [− 8.93, − 3.61], *p* < 0.001). Pain reduction was not significantly different between the orthoses and control groups during running (2.96, 95% CI [− 5.33, 11.24], *p* = 0.48). The I^2^s indicated considerable inconsistency in the resting (I^2^ = 88%) and running (I^2^ = 78%) categories. The 95% prediction interval was − 90,93 to 81,41 (supplementary data 4). The GRADE score for pain (in VAS) was rated as a low certainty score (supplementary data 5). Overall, a significant difference in pain reduction was found between the orthoses groups and the control groups (− 4.76, 95% CI [− 9.46, − 0.06], p0.05).

## Discussion

### Overall findings

The aim of this systematic review was to examine the effectiveness of orthoses for symptomatic flexible flatfeet in terms of patient-reported outcome measures compared to no orthoses or sham soles in children and adults. Three of the four included RCTs in children with no orthoses as control group found that improvements in PROMs were significantly higher in the orthoses than in the control groups [28, 30, 34]. The only study not to specifically include symptomatic participants did not find any differences between the groups [31]. Of the five included adult studies, two RCTs compared orthoses with sham soles and reported significantly higher PROM improvement in the orthoses group compared to the control group [34, 36]. The only prospective study, in which participants could choose between two different types of orthoses or an exercise program, noted improvement in all PROMs in all groups, with no significant differences observed between groups [35]. The remaining two adult RCTs also reported no significant difference between the intervention and control groups [32, 33]. The difference in effectiveness of orthoses reported by the included studies could not be explained by differences in study design, population, follow-up, or any of the other parameters presented in the studies. It is therefore difficult to draw any firm conclusions. This finding corresponds with two recently published reviews; in children, low to very low-certainty studies show that the effect of customized- or prefabricated foot orthoses on pain (in VAS), function and Health-Related Quality of Life is uncertain [41]. Whereas a systematic review on adults flatfoot also reported that no firm conclusions can be drawn on the effects and effectiveness of foot orthoses for adult patients [17]. Both studies did not specifically include studies with more than one measuring point. Moreover, flexible flatfeet was not a specific criterium [17]. Since foot orthoses probably have little effect on rigid flatfeet because of the limited range of motion, we think including specifically flexible flatfeet is an important criterium. However, the meta-analysis showed that the overall decrease in VAS score at follow-up compared to baseline was significantly higher in the orthoses group than the no-orthoses or sham sole group in adults. It is important to note that we used the DerSimonian and Laird random effects model for our meta-analysis, which is the only approach available in RevMan [39]. With this approach, confidence intervals are often slightly too narrow to encompass full uncertainty resulting from having estimated the degree of heterogeneity. There are alternative methods available such as the Hartung-Knapp-Sidik-Jonkman method, with better technical properties that may widen the confidence interval to reflect uncertainty in the estimation of between-study heterogeneity [42, 43]. The overall decrease may not be significant any more using these approaches. We also found a very broad 95% prediction interval. This can be largely explained by the small number of studies included in the meta-analysis. A prediction interval is considered more reliable in a meta-analysis with over ten studies. These findings confirm our conclusion that it is difficult to draw firm conclusions on the effectiveness of foot orthoses from the available data. This systematic review evaluated differences in PROMs between orthoses and control groups to assess effectiveness of orthoses from a patient perspective. Besides the nine studies included in this review, six prospective studies in children [44–49], one RCT [50], nine prospective [51–59] studies, and one retrospective study [60] in adults were identified which also described effectiveness of orthoses in terms of PROMs. However, these studies did not meet several of our inclusion criteria, i.e. the presence of a control group with no orthoses or sham soles and follow-up measurements. When control groups were present, they consisted of patients without symptomatic flexible flatfeet, with other orthoses, or with tape. In the orthoses groups of these studies, improvements in PROMs over time were seen in five child studies and eight adult studies. These results should however be interpreted with caution, since three studies in our systematic review showed significant improvements in PROMs in control groups as well, without significant differences between the orthoses and control groups [31, 32, 35].

Although child and adult flatfeet differ in etiology, all ages are included in this review because of the wide overlap in diagnosing flatfeet, type of symptoms, method of measurement, and treatment (Table 1).

### Strengths and limitations

This systematic review included all patient-reported outcomes of articles collecting information at baseline and follow-up in an orthoses group compared with a no orthoses or sham sole group in order to provide a reliable overview of the effectiveness of orthoses. The main limitation of the included studieswas the heterogeneity between included studies, which involved differences in patient characteristics, PROMs, conditions in which the PROM was measured, length of follow-up, and orthoses used, as well as the choice for sham sole or no soles as control group. The use of sham soles as a comparison group is questionable. The aim of using a sham sole in the control group is to decrease the psychological effect of the idea of being treated. However, it is important to be aware that a sham sole could have a positive influence on stability and thereby on PROMs. Another potential limitation to mention is ecological fallacy, specifically Simpson’s paradox. In this systematic review, descriptions are based on means of measurements, not individual patient data [61].

There are 40 definitions of flexible flatfeet in children [62]. As there is no universally accepted definition of flatfoot, studies investigating the effect of orthoses on flatfeet have conducted multiple diagnostic measurements based on physical examination and radiographs, which causes further heterogeneity [4, 8, 12, 13, 21, 63]. This article suggests three types of definitions for flatfeet to use in future research [62].

Only one RCT reported compliance and found that just half of the participants in the intervention group wore the orthoses most of the time or always, with lack of comfort as primary reason for not wearing them [33]. Besides compliance, activity and/or supported physiotherapy are also known to affect the PROMS of orthoses usage. Only one study controlled for physiotherapy as confounder with two extra groups [32].

### Future perspectives

For future studies, it is recommended to give extra consideration to subject characteristics, the control group used, and whether there is physiotherapy involved. Randomized controlled trials on the effectiveness of orthoses for flatfeet could likewise benefit from the use of a core outcome set for flatfoot trials [64] and/or a universal PROM tool. A promising possibility may be the use of Patient Reported Outcomes Measurement Information System (PROMIS) to provide evidence-based medicine in orthopedic clinics [65]. PROMIS offers a standardized tool to measure PROMs and allows for comparison of health outcomes across different disease states and populations regardless of age, culture, or disabilities.

## Conclusion

Based on the results of this systematic review we cannot conclude that foot orthoses are useful for flexible flatfoot in children and adults. However, the meta-analysis showed a significant decrease in pain in the adult orthoses group after treatment compared to the no-orthoses and sham orthoses groups.

## Supplementary Information

**Additional file 1.**

**Additional file 2.**

**Additional file 3.**

**Additional file 4.**

**Additional file 5.**

## Data Availability

The datasets used and/or analysed during the current study available from the corresponding author on reasonable request.

## References

[1] Pfeiffer M, Kotz R, Ledl T, Hauser G, Sluga M (2006). Prevalence of flat foot in preschool-aged children. Pediatrics..

[2] Michaudet C, Edenfield KM, Nicolette GW, Carek PJ (2018). Foot and Ankle Conditions: Pes Planus. FP Essent..

[3] Michaudet C, Edenfield KM, Nicolette GW, Carek PJ (2018). Foot and Ankle Conditions: Pes Planus. FP Essent.

[4] Evans AM, Rome K (2011). A Cochrane review of the evidence for non-surgical interventions for flexible pediatric flat feet. Eur J Phys Rehabil Med.

[5] Staheli LT (1987). Evaluation of planovalgus foot deformities with special reference to the natural history. J Am Podiatr Med Assoc.

[6] Uden H, Scharfbillig R, Causby R. The typically developing paediatric foot: how flat should it be? A systematic review. J Foot Ankle Res. 2017;10, 37. 10.1186/s13047-017-0218-1. 10.1186/s13047-017-0218-1 PMC5558233 28814975

[7] Dunn JE, Link CL, Felson DT, Crincoli MG, Keysor JJ, McKinlay JB (2004). Prevalence of foot and ankle conditions in a multiethnic community sample of older adults. Am J Epidemiol.

[8] Banwell HA, Mackintosh S, Thewlis D (2014). Foot orthoses for adults with flexible pes planus: a systematic review. J Foot Ankle Res..

[9] Hume P, Hopkins W, Rome K, Maulder P, Coyle G, Nigg B (2008). Effectiveness of foot orthoses for treatment and prevention of lower limb injuries : a review. Sports Med.

[10] Mills K, Blanch P, Chapman AR, McPoil TG, Vicenzino B (2010). Foot orthoses and gait: a systematic review and meta-analysis of literature pertaining to potential mechanisms. Br J Sports Med.

[11] Collins N, Bisset L, McPoil T, Vicenzino B (2007). Foot orthoses in lower limb overuse conditions: a systematic review and meta-analysis. Foot Ankle Int..

[12] Choi JY, Hong WH, Suh JS, Han JH, Lee DJ, Lee YJ (2020). The long-term structural effect of orthoses for pediatric flexible flat foot: A systematic review. Foot Ankle Surg..

[13] Hill M, Healy A, Chockalingam N (2020). Effectiveness of therapeutic footwear for children: A systematic review. J Foot Ankle Res..

[14] Kothari A, Dixon PC, Stebbins J, Zavatsky AB, Theologis T (2015). The relationship between quality of life and foot function in children with flexible flatfeet. Gait Posture.

[15] Kothari A, Stebbins J, Zavatsky AB, Theologis T (2014). Health-related quality of life in children with flexible flatfeet: A cross-sectional study. J Child Orthop.

[16] Kerr CM, Zavatsky AB, Theologis T, Stebbins J (2019). Kinematic differences between neutral and flat feet with and without symptoms as measured by the Oxford foot model. Gait Posture..

[17] Herchenröder M, Wilfling D, Steinhäuser J (2021). Evidence for foot orthoses for adults with flatfoot: a systematic review. J Foot Ankle Res..

[18] Spitzenverband Bund der Krankenkassen (GKV-Spitzenverband), 2. Bericht des GKV-Spitzenverbandes üder die Entwicklung der Mehrkostenvereinbarungen für Versorgungen mit Hilfsmittelleistungen. (2020).

[19] Desmyttere G, Hajizadeh M, Bleau J, Begon M (2018). Effect of foot orthosis design on lower limb joint kinematics and kinetics during walking in flexible pes planovalgus: A systematic review and meta-analysis. Clin Biomech (Bristol, Avon).

[20] Choi JY, Hong WH, Suh JS, Han JH, Lee DJ, Lee YJ (2020). The long-term structural effect of orthoses for pediatric flexible flat foot: A systematic review. Foot Ankle Surg.

[21] Dars S, Uden H, Banwell HA, Kumar S (2018). The effectiveness of non-surgical intervention (Foot Orthoses) for paediatric flexible pes planus: A systematic review: Update. PLoS One.

[22] Evans AM, Evans AM, Rome K, Carroll M, Hawke F (2022). Foot orthoses for treating paediatric flat feet. Cochrane Database Syst Rev.

[23] Gómez-Jurado I, Juárez-Jiménez JM, Munuera-Martínez PV (2021). Orthotic treatment for stage I and II posterior tibial tendon dysfunction (flat foot): A systematic review. Clin Rehabil.

[24] International Classification of Functioning, Disability and Health, 2022.

[25] Young J, Rowley L, Lalor S (2018). Use of Outcome Measures Among Prosthetists and Orthotists in the United Kingdom. JPO: Journal of Prosthetics and Orthotics.

[26] Young J, Rowley L, Lalor S, Cody C, Woolley H (2015). Measuring Change: an Introduction to Clinical Outcome Measures in Prosthetics and Orthotics.

[27] Page MJ, Moher D, Bossuyt PM, Boutron I, Hoffmann TC, Mulrow CD (2021). PRISMA 2020 explanation and elaboration: updated guidance and exemplars for reporting systematic reviews. BMJ..

[28] Asgaonkar B, Kadam P (2012). Effectiveness of valgus insole on pain, gait parameters and physiological cost index of walking in flat feet in 5-15 years. INDIAN J PHYSIOTHER OCCUP THER.

[29] Hsieh RL, Peng HL, Lee WC (2018). Short-term effects of customized arch support insoles on symptomatic flexible flatfoot in children: A randomized controlled trial. Medicine (Baltimore).

[30] Sinha S, Song HR, Kim HJ, Park MS, Yoon YC, Song SH (2013). Medial arch orthosis for paediatric flatfoot. JOrthopSurg(Hong Kong).

[31] Whitford D, Esterman A (2007). A randomized controlled trial of two types of in-shoe orthoses in children with flexible excess pronation of the feet. Foot Ankle Int.

[32] Andreasen J, Mølgaard CM, Christensen M, Kaalund S, Lundbye-Christensen S, Simonsen O (2013). Exercise therapy and custom-made insoles are effective in patients with excessive pronation and chronic foot pain--a randomized controlled trial. Foot (Edinb).

[33] Esterman A, Pilotto L (2005). Foot shape and its effect on functioning in Royal Australian Air Force recruits. Part 2: Pilot, randomized, controlled trial of orthotics in recruits with flat feet. Mil.Med..

[34] Shih YF, Wen YK, Chen WY (2011). Application of wedged foot orthosis effectively reduces pain in runners with pronated foot: a randomized clinical study. Clin.Rehabil..

[35] Taspinar O, Kabayel DD, Ozdemir F, Tuna H, Keskin Y, Mercimek OB (2017). Comparing the efficacy of exercise, internal and external shoe modification in pes planus: A clinical and pedobarographic study. J Back MusculoskeletRehabil.

[36] Yurt Y, Şener G, Yakut Y (2019). The effect of different foot orthoses on pain and health related quality of life in painful flexible flat foot: a randomized controlled trial. Eur.J.Phys.Rehabil.Med..

[37] Sterne JAC, Savović J, Page MJ, Elbers RG, Blencowe NS, Boutron I (2019). RoB 2: a revised tool for assessing risk of bias in randomised trials. BMJ..

[38] Schünemann HJ, Cuello C, Akl EA, Mustafa RA, Meerpohl JJ, Thayer K (2019). GRADE guidelines: 18. How ROBINS-I and other tools to assess risk of bias in nonrandomized studies should be used to rate the certainty of a body of evidence. J Clin Epidemiol.

[39] DerSimonian R, Laird N (1986). Meta-analysis in clinical trials. Control Clin Trials.

[40] Taylor C (2021). Range Rule for Standard Deviation.

[41] Evans AM, Rome K, Carroll M, Hawke F. Foot orthoses for treating paediatric flat feet. Cochrane Database Syst Rev. 2022. 10.1002/14651858.CD006311.pub3. 10.1002/14651858.CD006311.pub3 PMC8759438 35029841

[42] IntHout J, Ioannidis JPA, Borm GF (2014). The Hartung-Knapp-Sidik-Jonkman method for random effects meta-analysis is straightforward and considerably outperforms the standard DerSimonian-Laird method. BMC Med Res Methodol.

[43] Borenstein M (2019). Common mistakes in meta-analysis and how to avoid them.

[44] Dacharux W, Chadchavalpanichaya N (2017). The use of UCBL orthosis in patients with flatfoot in Foot Clinic, Siriraj Hospital. J.Med.Assoc.Thailand..

[45] Camurcu Y, Ucpunar H, Karakose R, Ozcan S, Sahin V (2021). Foot orthoses use for pediatric flexible flatfoot: comparative evaluation of quality of life for children and parents. J Pediatr Orthop B.

[46] Lee HJ, Lim KB, Yoo J, Yoon SW, Yun HJ, Jeong TH (2015). Effect of Custom-Molded Foot Orthoses on Foot Pain and Balance in Children With Symptomatic Flexible Flat Feet. Ann.Rehabil.Med..

[47] Mereday C, Dolan CM, Lusskin R (1972). Evaluation of the University of California Biomechanics Laboratory shoe insert in "flexible" pes planus. Clin Orthop Relat Res.

[48] Pauk J, Ezerskiy V (2011). The Effect of Foot Orthotics on Arch Height: Prediction of Arch Height Correction in Flat-foot Children. Biocybernetics and Biomedical Engineering.

[49] Pandey S, Pal CP, Kumar D, Singh P (2013). Flatfoot in Indian population. J Orthop Surg (Hong Kong).

[50] Xu R, Wang Z, Ren Z, Ma T, Jia Z, Fang S (2019). Comparative Study of the Effects of Customized 3D printed insole and Prefabricated Insole on Plantar Pressure and Comfort in Patients with Symptomatic Flatfoot. Med.Sci.Monit..

[51] Açak M (2020). The effects of individually designed insoles on pes planus treatment. Sci.Rep..

[52] Bek N, Öznur A, Kavlak Y, Uygur F (2003). The effect of orthotic treatment of posterior tibial tendon insufficiency on pain and disability, The. Pain Clinic.

[53] Gijon-Nogueron G, Cortes-Jeronimo E, Cervera-Marin JA, Diaz-Mohedo E, Lopezosa-Reca E, Fernandez-Sanchez M (2015). The effects of custom-made foot orthosis using the Central Stabilizer Element on foot pain. Prosthetics Orthot Int.

[54] Karthikeyan J, Singh K, Govind S, Mahalingam K, Vamsi S, Annamalai P (2020). To compare the effectiveness of taping and arch support on the flexible flat foot on a random population, Indian J. Forensic. Med.Toxicol..

[55] Nowacki RM, Air ME, Rietveld AB (2013). Use and effectiveness of orthotics in hyperpronated dancers. J Dance MedSci.

[56] Stell JF, Buckley JG (1998). Controlling excessive pronation: A comparison of casted and non-casted orthoses. Foot..

[57] Motimath B, Parveen S, Chivate D (2019). A Comparison between Kinesio Taping and Medial Arch Support Combined with Exercises in Adult Flatfoot -- An Experimental Study. INDIAN J PHYSIOTHER OCCUP THER..

[58] Kumar AAU. A Study to Analyse the Effectiveness of Physical Therapy and Wedged Foot Orthotic Devices on Pain in Runners with Pronated Foot. Medicine. 2020;99.

[59] Zammit GV, Payne CB (2007). Relationship between positive clinical outcomes of foot orthotic treatment and changes in rearfoot kinematics. J Am Podiatr Med Assoc.

[60] Nielsen MD, Dodson EE, Shadrick DL, Catanzariti AR, Mendicino RW, Malay DS (2011). Nonoperative care for the treatment of adult-acquired flatfoot deformity. J. Foot Ankle Surg..

[61] Rücker G, Schumacher M (2008). Simpson's paradox visualized: The example of the Rosiglitazone meta-analysis. BMC. Med Res Methodol.

[62] Banwell HA, Paris ME, Mackintosh S, Williams CM (2018). Paediatric flexible flat foot: how are we measuring it and are we getting it right? A systematic review. J Foot Ankle Res..

[63] Desmyttere G, Hajizadeh M, Bleau J, Begon M (2018). Effect of foot orthosis design on lower limb joint kinematics and kinetics during walking in flexible pes planovalgus: A systematic review and meta-analysis. Clin Biomech (Bristol, Avon).

[64] Webbe J, Sinha I, Gale C (2018). Core Outcome Sets. Arch Dis Child Educ Pract Ed.

[65] Bertani A, Cappello A, Benedetti MG, Simoncini L, Catani F (1999). Flat foot functional evaluation using pattern recognition of ground reaction data. Clin Biomech (Bristol, Avon).

