# Supplementary material for: Foot orthoses for flexible flatfeet in children and adults: a systematic review and meta-analysis of patient-reported outcomes
Source: BMC Musculoskelet Disord. 2023 Jan 7;24:16. doi: 10.1186/s12891-022-06044-8 (PMC9825043; doi:10.1186/s12891-022-06044-8)
Supplement: Supplementary file 1 — Additional file 1. [file 12891_2022_6044_MOESM1_ESM.docx]

**Supplementary data 1:** Search strategy per database

Search strategy in Pubmed on 19 February 2021: 1158 results

(Flatfoot[Mesh] OR "flat foot" OR "Talipes Valgus" OR Splayfoot OR "Flat Feet" OR "Flatfeet" OR "Pes Planus" OR planovalgus OR "pes plan*" OR "Acquired Adult Flatfoot Deformity" OR "Flexible Flatfoot" OR "flat footedness" OR "longitudinal arch" OR "fallen arch" OR "pronat*") AND (Foot Orthoses[Mesh] OR "foot orthos*" OR "Foot Orthotic Device*" OR "Foot Arch Support*" OR "Orthotic Shoe Insert*" OR "orthoses" OR "Orthotic Insole*" OR orthotics OR "foot orthotic treatment" OR "arch support" OR "arch insert" OR "shoe insert" OR inserts OR "shoe insole" OR insoles OR soles OR sole OR inlays OR inlay)

Search strategy in OVID/Medline on 22 February 2021: 564 results
(exp Flatfoot/ OR flat foot OR Talipes Valgus OR Splayfoot OR Flat Feet OR Flatfeet OR Pes Planus OR planovalgus OR pes plan* OR Acquired Adult Flatfoot Deformity OR Flexible Flatfoot OR flat footedness OR longitudinal arch OR fallen arch OR pronat*) AND (exp Foot Orthoses/ OR foot orthos* OR Foot Orthotic Device* OR Foot Arch Support* OR Orthotic Shoe Insert* OR orthoses OR Orthotic Insole* OR orthotics OR foot orthotic treatment OR arch support OR arch insert OR shoe insert OR inserts OR shoe insole OR insoles OR soles OR sole OR inlays OR inlay)

Search strategy in Embase on 24 February 2021: 1019 results

('Flatfoot'/exp OR "flat foot" OR "Talipes Valgus" OR Splayfoot OR "Flat Feet" OR Flatfeet OR "Pes Planus" OR planovalgus OR "pes plan*" OR "Acquired Adult Flatfoot Deformity" OR "Flexible Flatfoot" OR "flat footedness" OR "longitudinal arch" OR "fallen arch" OR pronat*) AND ('Foot Orthoses'/exp OR "foot orthos*" OR "Foot Orthotic Device*" OR "Foot Arch Support*" OR "Orthotic Shoe Insert*" OR orthoses OR "Orthotic Insole*" OR orthotics OR "foot orthotic treatment" OR "arch support" OR "arch insert" OR "shoe insert" OR inserts OR "shoe insole" OR insoles OR soles OR sole OR inlays OR inlay)

Search strategy in Web-Of-Science on 1 March 2021: 528 results

TI=(Flatfoot OR "flat foot" OR "Talipes Valgus" OR Splayfoot OR "Flat Feet" OR Flatfeet OR "Pes Planus" OR planovalgus OR "pes plan*" OR "Acquired Adult Flatfoot Deformity" OR "Flexible Flatfoot" OR "flat footedness" OR "longitudinal arch" OR "fallen arch" OR pronat*) AND TI=("Foot Orthoses" OR "foot orthos*" OR "Foot Orthotic Device*" OR "Foot Arch Support*" OR "Orthotic Shoe Insert*" OR orthoses OR "Orthotic Insole*" OR orthotics OR "foot orthotic treatment" OR "arch support" OR "arch insert" OR "shoe insert" OR inserts OR "shoe insole" OR insoles OR soles OR sole OR inlays OR inlay)

Search strategy in CINAHL on 1 March 2021: 596 results

#1=TI=(Flatfoot OR "flat foot" OR "Talipes Valgus" OR Splayfoot OR "Flat Feet" OR Flatfeet OR "Pes Planus" OR planovalgus OR "pes plan*" OR "Acquired Adult Flatfoot Deformity" OR "Flexible Flatfoot" OR "flat footedness" OR "longitudinal arch" OR "fallen arch" OR pronat*) #2=TI=("Foot Orthoses" OR "foot orthos*" OR "Foot Orthotic Device*" OR "Foot Arch Support*" OR "Orthotic Shoe Insert*" OR orthoses OR "Orthotic Insole*" OR orthotics OR "foot orthotic treatment" OR "arch support" OR "arch insert" OR "shoe insert" OR inserts OR "shoe insole" OR insoles OR soles OR sole OR inlays OR inlay) 
#3=#2 AND #1 
#4=AB=(Flatfoot OR "flat foot" OR "Talipes Valgus" OR Splayfoot OR "Flat Feet" OR Flatfeet OR "Pes Planus" OR planovalgus OR "pes plan*" OR "Acquired Adult Flatfoot Deformity" OR "Flexible Flatfoot" OR "flat footedness" OR "longitudinal arch" OR "fallen arch" OR pronat*) 
#5=AB=("Foot Orthoses" OR "foot orthos*" OR "Foot Orthotic Device*" OR "Foot Arch Support*" OR "Orthotic Shoe Insert*" OR orthoses OR "Orthotic Insole*" OR orthotics OR "foot orthotic treatment" OR "arch support" OR "arch insert" OR "shoe insert" OR inserts OR "shoe insole" OR insoles OR soles OR sole OR inlays OR inlay) 
#6=#5 AND #4 
#7=#6 OR #3

Search stategy in Cochrane on 1 March 2021: 116 results

#1=Mesh [flatfoot]

#2="flat next foot" OR "Talipes next Valgus" OR Splayfoot OR "Flat next Feet" OR Flatfeet OR "Pes next Planus" OR planovalgus OR "pes next plan*" OR "Acquired next Adult next Flatfoot next Deformity" OR "Flexible next Flatfoot" OR "flat next footedness" OR "longitudinal next arch" OR "fallen next arch" OR pronat*

#3=Mesh [foot orthoses]

#4="foot next orthos*" OR "Foot next Orthotic next Device*" OR "Foot next Arch next Support*" OR "Orthotic next Shoe next Insert*" OR orthoses OR "Orthotic next Insole*" OR orthotics OR "foot next orthotic next treatment" OR "arch next support" OR "arch next insert" OR "shoe next insert" OR inserts OR "shoe next insole" OR insoles OR soles OR sole OR inlays OR inlay

#5=(#1 OR #2) AND (#3 OR #4)

Search strategy in Scopus on 3 March 2021: 648 results

(INDEXTERMS("Flatfoot") OR {flat foot} OR {Talipes Valgus} OR Splayfoot OR {Flat Feet} OR Flatfeet OR {Pes Planus} OR planovalgus OR {pes plan*} OR {Acquired Adult Flatfoot Deformity} OR {Flexible Flatfoot} OR {flat footedness} OR {longitudinal arch} OR {fallen arch} OR pronat*) AND (INDEXTERMS("Foot Orthoses") OR {foot orthos*} OR {Foot Orthotic Device*} OR {Foot Arch Support*} OR {Orthotic Shoe Insert*} OR orthoses OR {Orthotic Insole*} OR orthotics OR {foot orthotic treatment} OR {arch support} OR {arch insert} OR {shoe insert} OR inserts OR {shoe insole} OR insoles OR soles OR sole OR inlays OR inlay) AND  ( LIMIT-TO ( DOCTYPE ,  "re" ) )  AND  ( LIMIT-TO ( SUBJAREA ,  "MEDI" ) )
