# Supplementary material for: Foot orthoses for flexible flatfeet in children and adults: a systematic review and meta-analysis of patient-reported outcomes
Source: BMC Musculoskelet Disord. 2023 Jan 7;24:16. doi: 10.1186/s12891-022-06044-8 (PMC9825043; doi:10.1186/s12891-022-06044-8)
Supplement: Supplementary file 2 — Additional file 2. [file 12891_2022_6044_MOESM2_ESM.docx]

**Supplementary data 2:** Reason for exclusion

| Acak 2020 | No control group |
| --- | --- |
| Bek 2003 | No control group |
| Gijon 2015 | The control group had no flatfeet |
| Karthikeyan 2020 | No patient-reported outcome at baseline |
| Kulig 2009 | Included PTTD stage 1, when there is not yet a fallen arch, this is not a flatfoot |
| Kumar 2019 | Follow-up time was unclear |
| Mothimath 2019 | The control group had tape |
| Nowacki 2013 | Prospective design: No control group  Retrospective design: This design was excluded in the current review |
| Nielsen 2011 | No patient-reported outcome at baseline |
| Stell 1998 | No control group without orthoses, two types of orthoses are investigated |
| Xu 2019 | No control group without orthoses, two types of orthoses are investigated |
| Zammit 2007 | No control group without orthoses, two types of orthoses are investigated |

Reference list of all studies about adults that underwent full-text review but were excluded

| Camurcu 2021 | No patient-reported outcome at baseline |
| --- | --- |
| Dacharux 2018 | First patients were included, when patients did not were the orthoses, the patients were excluded from the study |
| Lee 2015 | No control group |
| Mereday 1972 | No control group |
| Pandey 2013 | The control group had no flatfeet |
| Pauk 2011 | The control group had no flatfeet |

Reference list of all studies about children that underwent full-text review but were excluded
