# Supplementary material for: Foot orthoses for flexible flatfeet in children and adults: a systematic review and meta-analysis of patient-reported outcomes
Source: BMC Musculoskelet Disord. 2023 Jan 7;24:16. doi: 10.1186/s12891-022-06044-8 (PMC9825043; doi:10.1186/s12891-022-06044-8)
Supplement: Supplementary file 3 — Additional file 3. [file 12891_2022_6044_MOESM3_ESM.docx]

**Supplementary data 3:** Drop-outs

|  | **First author, year published** | **N** | **Orthoses versus control group (N)** | **FU** | **Total drop-outs per group; reason for drop-out per group (N)*** | **Extra information / reasons for drop-out** |
| --- | --- | --- | --- | --- | --- | --- |
| Children | Asgaonkar 2012 [34] | 60 | I: Valgus pad, rubber (30)  C: No orthoses (30) | 1Y | I: 15  C: 5 | 80 subjects started the study, drop-outs were excluded  Reasons: 1. irregular users, 2. Left the school |
|  | Hsieh 2018 [42] | 52 | I: Thermoplastic insoles (26)  C: No orthoses (26) | 3M | I: 4; Lost to follow up (2) Due to parental time reason (2)  C: 0 |  |
|  | Sinha 2013 [37] | 81 | I: Medial arch orthoses (55)  C: No orthoses (26) | >2Y | I: NA  C: NA | 101 subjects were evaluated, 20 patients lost to follow-up or non-compliant to the treatment were excluded |
|  | Whitford 2007 [39] | 160 | I: Custom orthoses (52)  I: Ready-made orthoses (54)  C: No orthoses (54) | 1Y | I: 7  I: 5  C: 6 | Parental participation burden and loss of contact. Reasons for dropping out in each group were similar |
| Adults | Andreasen 2013 [35] | 70 | I: Medial arch and calcaneus support (20)  I: Exercise program (19)  I: Orthoses + exercise program (17)  C: Folder with exercises (14) | 1Y | I: 0  I: 1; Time issues (1)  I: 3; Time issues (1) Other disease (2)  C: 6; Time issues (4) Other disease (1) Abroad (1) | Time issues were related to motivation and work. Other reasons for drop-out were diseases as cancer, depression, and stress. |
|  | Esterman 2005 [38] | 47 | I: Flexible/semi-rigid orthoses (25)  C: No orthoses (22) | 8W | I: 0  C: 0 | Of the 25 recruits provided with orthotics, 20 provided details of frequency of use. Only 10 of the 20 wore the orthotics all of most of the time. |
|  | Shih 2011 [36] | 24 | I: Wedged foot orthoses, EVA (12)  C: Sham orthoses (12) | 2W | I: 0  C: 0 |  |
|  | Taspinar 2017 [41] | 60 | I: Medial arch support (20)  I: Thomas heel, external support (20)  C: Exercise program (20) | 3M | I: 0  I: 0  C: 0 |  |
|  | Yurt 2019 [40] | 67 | I: CAD-CAM orthoses and exercise (22)  I: Conventional orthoses and exercise (22)  C: Sham orthoses and exercise (23) | 8W | I: 1; Lost to follow-up, we could not contact (1)  I: 1; Lost to follow-up, we could not contact (1)  C: 2; Lost to follow-up, we could not contact (2) |  |

| *Groups are listed in the same order as column ‘Orthoses versus control group (N)’  Abbreviations: I = intervention group; C = control group |
| --- |
