# Supplementary material for: Foot orthoses for flexible flatfeet in children and adults: a systematic review and meta-analysis of patient-reported outcomes
Source: BMC Musculoskelet Disord. 2023 Jan 7;24:16. doi: 10.1186/s12891-022-06044-8 (PMC9825043; doi:10.1186/s12891-022-06044-8)
Supplement: Supplementary file 4 — Additional file 4. [file 12891_2022_6044_MOESM4_ESM.docx]

**Supplementary data 4:** Prediction interval
