# Supplementary material for: Foot orthoses for flexible flatfeet in children and adults: a systematic review and meta-analysis of patient-reported outcomes
Source: BMC Musculoskelet Disord. 2023 Jan 7;24:16. doi: 10.1186/s12891-022-06044-8 (PMC9825043; doi:10.1186/s12891-022-06044-8)
Supplement: Supplementary file 5 — Additional file 5. [file 12891_2022_6044_MOESM5_ESM.docx]

**Supplementary data 5:** Grade score

**Author(s):** Leonoor N.T. Oerlemans, MD, Charles M.M. Peeters, MD, Roelina Munnik-Hagewoud, PhD, Ingrid M. Nijholt, PhD, Adhiambo Witlox, MD, PhD, Cees C.P.M. Verheyen, MD, PhD

**Question:** The effect of orthoses versus no orthoses for patients with flexible flatfeet

| **Certainty assessment** | | | | | | | **№ of patients** | | **Effect** | | **Certainty** | **Importance** |
| --- | --- | --- | --- | --- | --- | --- | --- | --- | --- | --- | --- | --- |
| **№ of studies** | **Study design** | **Risk of bias** | **Inconsistency** | **Indirectness** | **Imprecision** | **Other considerations** | **Orthoses** | **No orthoses** | **Relative (95% CI)** | **Absolute (95% CI)** |  |  |
| **Pain (follow-up: range 2 weeks to 1 years; assessed with: VAS scale; Scale from: 0 to 10)** | | | | | | | | | | | | |
| 3 | randomised trials | serious^a^ | not serious | not serious | serious^b^ | none | 167 | 157 | - | MD **4.76 VAS lower** (9.46 lower to 0.06 lower) | ⨁⨁◯◯ Low | CRUCIAAL |

**CI:** confidence interval; **MD:** mean difference

#### Explanations

a. Methodological quality of the studies was assessed using the Revised Cochrane risk-of-bias tool for randomized trials (RoB 2) and the Risk Of Bias In Non-Randomized Studies of Interventions (ROBINS-I). The quality assessment showed that the risk of bias was serious (Figure 2).

b. The reason for rating inconsistency serious is mainly based on relatively large amount of drop-outs of participants and non-compliance; participants not wearing the orthoses in the orthoses groups and participants wearing orthoses in the no orthoses groups.
